# Multidisciplinary Perspective of Spread Through Air Spaces in Lung Cancer: A Narrative Review

**DOI:** 10.3390/cancers17203374

**Published:** 2025-10-19

**Authors:** Riccardo Orlandi, Lorenzo Bramati, Maria C. Andrisani, Giorgio A. Croci, Claudia Bareggi, Simona Castiglioni, Francesca Romboni, Sara Franzi, Davide Tosi

**Affiliations:** 1 Department of Thoracic Surgery, University of Milan, 20122 Milan, Italy; 2 Thoracic Surgery and Lung Transplantation Unit, Fondazione IRCCS Ca’ Granda—Ospedale Maggiore Policlinico of Milan, 20122 Milan, Italydavide.tosi@policlinico.mi.it (D.T.); 3 Department of Radiology, Fondazione IRCCS Ca’ Granda—Ospedale Maggiore Policlinico of Milan, 20122 Milan, Italy; 4 Pathology Unit, Fondazione IRCCS Ca’ Granda—Ospedale Maggiore Policlinico of Milan, 20122 Milan, Italy; 5 Oncology Unit, Fondazione IRCCS Ca’ Granda—Ospedale Maggiore Policlinico of Milan, 20122 Milan, Italy; 6 Radiotherapy Unit, IRCCS Policlinico San Donato, 20097 Milan, Italy

**Keywords:** non-small cell lung cancer, STAS, surgery, adjuvant treatment, prognosis

## Abstract

Spread Through Air Spaces (STAS) is an emerging pattern of tumor invasion in lung cancer, first recognized by the World Health Organization in 2015. This narrative review examines STAS from a multidisciplinary perspective, integrating pathologic, radiologic, oncologic, and surgical points of view, together with molecular biology to assess its clinical significance, diagnostic challenges, and therapeutic implications. Pathologically, STAS is characterized by tumor cells floating beyond the main tumor, contributing to recurrence and poor prognosis. Radiologic advancements suggest potential imaging markers for STAS, such as spiculation, the absence of an air bronchogram, solid tumor components, as well as high fluorodeoxyglucose uptake, though definitive preoperative identification remains challenging. Oncologic studies link STAS to aggressive tumor behavior and lympho-vascular invasion, suggesting a role for adjuvant chemotherapy even in the earliest stages of disease; furthermore, specific molecular alterations have been discovered, including EGFR wild-type status and ALK/ROS1 rearrangements together with high Ki-67 expression, tumor necrosis, and alterations in cell adhesion proteins like E-cadherin. Surgical aspects highlight the increased risk of recurrence following limited resection, raising concerns about optimal surgical strategies. The debate over STAS as a true invasion mechanism versus an artifact from surgical handling underscores the need for standardized pathological evaluation. This review aims to refine STAS detection, integrate it into multidisciplinary treatment decision-making, and assess its potential as a staging criterion in lung cancer management.

## Simple Summary

Spread Through Air Spaces (STAS) is a recently recognized pattern of lung cancer invasion where tumor cells spread into airways beyond the main tumor. This review explores STAS from multiple angles—pathology, radiology, surgery, oncology, and molecular biology. While the exact cause of STAS is still unclear, evidence points to biological processes like EMT (epithelial–mesenchymal transition), immune system changes, and certain genetic markers. Frozen section analysis still has the limitation of low sensitivity and cannot fully meet clinical needs. Imaging tools—especially CT scans and radiomic models—are showing promise in predicting STAS before surgery. STAS has been found not just in adenocarcinomas, but also in squamous cell carcinomas, neuroendocrine tumors, and lung metastases from other cancers. In many cases, STAS-positive patients benefit more from lobectomy over limited resection and may require adjuvant chemotherapy. Overall, STAS is an important factor in lung cancer management, and ongoing research aims to improve its detection and guide more personalized treatment.

## 1. Introduction

Lung cancer remains the leading cause of cancer-related mortality worldwide, responsible for millions of deaths each year [1]. In 2015, the concept of spread through air spaces (STAS) was first introduced by Kadota and colleagues [2] and then recognized by the World Health Organization as a distinct invasion pattern in lung adenocarcinoma [3]. STAS is defined by the presence of tumor cells within alveolar spaces beyond the main tumor margin, indicating a mode of dissemination distinct from traditional routes such as lymphatic, vascular, or direct invasion. The identification of STAS has reshaped the understanding of lung cancer progression and significantly influenced tumor classification, prognostic evaluation, and surgical planning. It is now recognized as a key risk factor for locoregional recurrence and poor survival outcomes, particularly in patients undergoing limited resections like segmentectomy or wedge resection [4]. While initially documented in adenocarcinoma, STAS has since been observed in other histological subtypes [5]—including squamous cell carcinoma, small cell lung cancer, and pleomorphic carcinoma—highlighting its potential as a more universal mechanism of tumor spread. Increasing attention is being directed toward uncovering the biological mechanisms underlying STAS and developing predictive models to guide clinical decision-making. However, no standardized, clinically validated model—based on either pathological or radiological criteria—has been established. As interest grows in limited resection techniques for early-stage lung cancer, a clearer understanding of STAS is critical for optimizing treatment strategies and improving patient outcomes. A comprehensive literature search was conducted across PubMed, Scopus, and Web of Science databases for studies published between January 2015 and June 2025. The following search terms were used in various combinations: ‘spread through air spaces,’ ‘STAS,’ ‘lung cancer,’ ‘non-small cell lung cancer,’ ‘adenocarcinoma,’ and ‘surgery.’ Only English-language articles were included. We considered original research articles, reviews, and meta-analyses addressing pathological, radiological, surgical, oncological, and molecular aspects of STAS. Case reports, conference abstracts, and studies lacking histopathological confirmation of STAS were excluded. The objective of this review is to integrate insights from pathology, radiology, surgery, and oncology to provide a comprehensive multidisciplinary assessment of STAS and its implications for clinical practice.

## 2. Pathogenesis

Since the introduction of the STAS concept, extensive research has been conducted to understand its biology. However, the precise mechanisms behind its development remain unclear [6], and current insights are based largely on preliminary data and theoretical models. Epithelial–mesenchymal transition (EMT) plays a key role in STAS development by enabling epithelial tumor cells to acquire motility and detach from their surroundings [7]. Markers of EMT, including decreased E-cadherin and increased vimentin and Ki-67 expression, were reported to be associated with STAS [8]. Furthermore, a biological link between EMT and STAS is testified by the increased rate of STAS in neoplasms showing nuclear β-catenin—a hallmark of EMT activation [9]. Matrix metalloproteinase-7 (MMP-7) has also been implicated [10]: MMP-7 promotes tumor cell detachment by degrading extracellular matrix components, facilitating their release into alveolar spaces and eventual reattachment to nearby structures. Several studies have explored how the tumor microenvironment influences STAS. STAS was found to be associated with high levels of α-smooth muscle actin (α-SMA)-positive cancer-associated fibroblasts and CD204-positive tumor-associated macrophages (TAMs) [11]; a strong correlation between STAS and CD68-positive TAMs was also noted [12]. These results suggest that macrophages and regulatory T cells may promote the development of STAS. In addition, several studies have reported a link between STAS and increased expression of programmed death-ligand 1 (PD-L1) in tumor and stromal cells [13], suggesting that immune evasion might play a role. Molecular profiling has revealed associations between STAS and certain genetic backgrounds. STAS was found to be more common in tumors with wild-type EGFR and in those harboring ALK rearrangements in an Asian cohort study [14], though a European study [15] reported no significant association between STAS and EGFR or KRAS status, indicating potential geographic or cohort-specific variability. Indeed, these conflicting findings may reflect differences in patient populations, such as ethnic and geographic backgrounds, as genetic alterations in lung cancer vary notably across regions. On the other hand, methodological factors, such as the sensitivity of molecular testing and criteria for defining STAS, might also explain the variability observed between studies. Further research using standardized methodologies and diverse cohorts is needed to clarify the relationship between STAS and molecular alterations. The possibility that STAS may sometimes be an artifact introduced during specimen handling—rather than a true in vivo phenomenon—has been the subject of debate. Some studies suggest that tumor cells may be displaced by surgical tools (“spread through a knife surface,” or STAKS), particularly in lung tissue with loose alveolar architecture [16]. This raises the concern that STAS-like patterns could arise artificially during specimen sectioning. Despite this, other investigations found no significant difference in STAS detection between freshly cut and fixed samples, arguing against the artifact theory [17]. No significant difference in STAS prevalence between surgical approaches was found [18], suggesting that manipulation and compression during minimally invasive surgery do not increase STAS rate. Some researchers proposed that artificially induced STAS may have distinct morphological features—such as irregular tumor clusters, linear bands of cells, or isolated cells beyond the alveolar framework still attached to the primary tumor [19]. The development of STAS appears to be influenced by a complex interplay of EMT-related changes, microenvironmental factors, genetic alterations, and possibly technical artifacts. While our understanding has grown, definitive mechanisms and clinical implications require further investigation.

## 3. Radiologic Perspective

**Figure 1:**
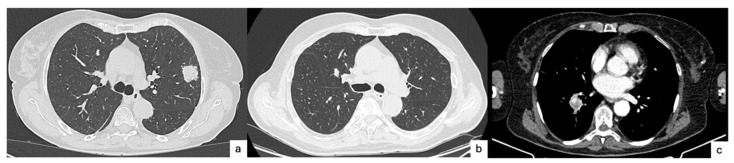
Three cases of STAS-positive lung cancers, as they appeared on CT scan. (**a**). Left upper lobe lung lesion presenting as solid lobulated nodule undergoing lobectomy, later revealed to be invasive lung adenocarcinoma pT2a pN0, STAS-positive. (**b**). Right upper lobe lung lesion presenting as solid lobulated nodule undergoing lobectomy, later revealed to be invasive lung squamous cell carcinoma pT1b pN0, STAS-positive. (**c**). Right lower lobe lung lesion presenting as solid nodule with central low attenuation (mediastinal window) undergoing lobectomy, later revealed to be invasive lung adenocarcinoma pT2a pN0, STAS-positive.

Given the critical role STAS plays in surgical decision-making, researchers have increasingly turned to imaging as a non-invasive method to predict STAS preoperatively. CT findings for predicting the presence of STAS, according to recent studies, include central low attenuation, lobulation and percentage of the solid component [20,21]. Figure 1a,b presents two cases of STAS-positive lung cancers, appearing as solid, lobulated nodules with central low attenuation.

**Figure 2:**
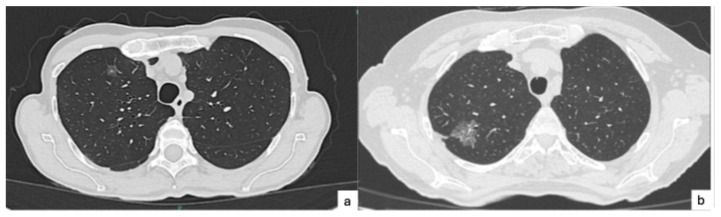
Two cases of STAS-negative lung cancers, as they appeared on CT scan. (**a**). Right upper lobe lung lesion presenting as ground-glass opacity with central solid component undergoing segmentectomy, later revealing to be invasive lung adenocarcinoma pT1a pN0, STAS-negative. (**b**). Right upper lobe lung lesion presenting as ground-glass opacity with central solid component undergoing lobectomy, later revealing to be invasive lung adenocarcinoma pT2a pN0, STAS-negative.

Several studies have shown that percentage of the solid component is better in predicting STAS than the maximal diameter of the solid component. Central low attenuation is defined as an area of low attenuation within a solid or part-solid lesion using the mediastinal window setting. Lobulation is the descriptive term referring to a lobule-like and often asymmetric protrusion at the margins of a structure. Other common malignant signs, such as spiculation, air bronchogram, pleural indentation, and vascular convergence signs, also have some predictive value in STAS, especially in invasive lung adenocarcinoma [22]. The presence of ground-glass opacity (GGO) is not a predictive factor of STAS; rather, it is negatively related to STAS-positive adenocarcinoma [23]. Figure 2a,b presents two cases of STAS-negative lung cancers, appearing as sub-solid lesions.

STAS is reported to occur in about 2% of pure GGO lesions and in 10% of GGO-predominant lesions [24], and roughly three times more often in pure solid nodules [21]. These findings suggested that CT imaging could help identify STAS-positive patients before surgery and guide surgical planning accordingly. PET-CT findings frequently reveal an increased maximum standardized uptake value (SUV), which correlates with a higher probability of STAS [25]: SUVmax, rather than metabolic tumor volume or total lesion glycolysis, has been identified as a more reliable indicator for predicting STAS, though a precise cut-off cannot be identified. Radiomics—the extraction of high-dimensional quantitative features from radiological images—has become a powerful tool in cancer diagnostics and prognosis. Several studies have explored its potential in predicting STAS. However, the diversity of models and study designs has made this field challenging to summarize. CT-based radiomics models have been widely explored for preoperative STAS prediction. PyRadiomics developed a Naïve Bayes model based on five key radiomic features (sphericity, 90th percentile, grey level length variance, cluster tendency, grey level zone variance), achieving AUCs of 0.63 (internal validation) and 0.69 (external validation) [26]. A random forest algorithm using 12 features improved performance, with an AUC of 0.754 [27]. Peritumoral radiomics models, which analyze the tissue immediately surrounding the tumor, outperformed traditional models with AUCs of 0.76 and 0.70 in validation sets [28]. Combined models that integrate tumoral and peritumoral radiomic signatures achieved higher accuracy, with AUCs of 0.854 and 0.870 in development and validation cohorts, respectively [29]. More sophisticated volumetric approaches, such as the GPTV10 model, which includes different peritumoral volumes, further increased discriminative power with AUCs of 0.887 (training), 0.876 (internal validation), and 0.868 (external validation) [30]. Deep learning approaches applied to CT data have also shown promise. A deep-learning model focusing on solid component gating in ground-glass-predominant adenocarcinomas achieved an AUC of 0.82, demonstrating effectiveness in guiding surgical decisions for these patients [31]. PET/CT-based models incorporate metabolic parameters in addition to morphologic features and were designed to overcome the limitations of CT models. One PET/CT radiomic model using SUVmax, consolidation-to-tumor ratio, and lobulation sign yielded AUCs of 0.796 and 0.821 in training and testing cohorts [32]. The Age-Density-SUVmax model, based on 18F-FDG PET/CT and logistic regression, delivered AUCs of 0.808, 0.786, and 0.806 across three datasets [33]. Spectral CT, another emerging modality, identified quantitative parameters such as CT100keVa and CT100keVv as independent predictors of STAS [34]. When these spectral parameters were combined with standard CT features, the model achieved an AUC of 0.952, with 90.6% sensitivity and 86.7% specificity. Despite these advances, the clinical translation of radiomics and deep learning models remains limited. Barriers include the need for large, diverse, and well-annotated datasets for training and validation; lack of standardized imaging protocols and feature extraction methods; and the complexity of integrating these models into routine preoperative workflows. Additionally, many of the current models have been developed and validated retrospectively in single-center cohorts, raising concerns about generalizability. Interpretability of deep learning models and regulatory approval pathways are further challenges. Overcoming these hurdles will require multicenter prospective studies, harmonization of imaging and analysis protocols, and close collaboration between clinicians, radiologists, data scientists, and regulatory bodies to ensure that these promising tools can be reliably and safely implemented in clinical practice.

## 4. Pathologic Perspective

**Figure 3:**
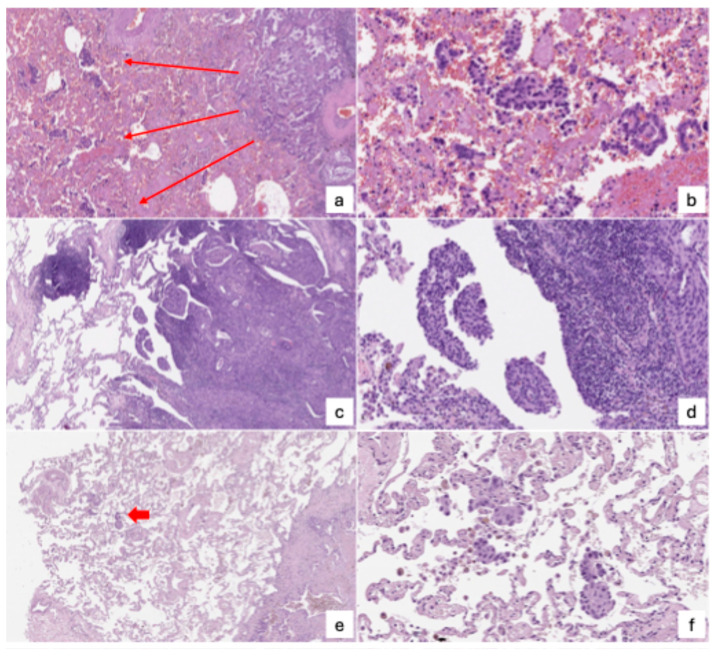
Different morphologies of STAS as appear under microscope. Case 1 ((**a**), H/E, 40×; (**b**), H/E, 200×) depicts a clear-cut STAS, featuring continuous single cell and papillary type spread from the edge of invasive adenocarcinoma (red arrows). Case 2 ((**c**), H/E, 40×; (**d**), H/E, 200×) depicts a case that fulfills WHO criteria but would pose great disagreement among pathologists: nests of atypical cells are observed detached to the edge of the tumor, with no intervening, spared alveoli, and with dubious presence of ciliated, non-neoplastic cells. Case 3 ((**e**), H/E, 40×; (**f**), H/E, 200×) still represents a critical instance, as a single cluster of atypical cells (red arrow) is observed at a distance from the tumor front, and close to the edge of the slide: as the WHO criteria are not fulfilled, pathologists in daily practice may disagree.

As early as 2000, the idea of aerogenous spread was introduced [35], describing it as tumor cells freely present within the alveolar spaces. Building on this, small papillary clusters within bronchoalveolar spaces adjacent to lung adenocarcinomas were later identified [36]. In 2013, the term “tumor island” was coined [37] to describe dense clusters of cancer cells found within alveolar cavities, which were associated with worse outcomes following resection in early-stage lung adenocarcinoma. The concept of STAS was formally introduced in 2015 [2], defined as the migration of tumor cells into alveolar spaces surrounding the main tumor. That same year, the World Health Organization acknowledged STAS as a distinct mode of invasion [3]. It has also been recommended as a histological descriptor in the ninth edition of the TNM staging system for lung cancer [38]. Morphologically, STAS may present in three main forms, as presented in Figure 3:

- Micropapillary clusters—papillary structures without a central fibrovascular core, sometimes forming ring-like shapes within alveolar spaces.
- Solid nests or tumor islands—compact groups of tumor cells occupying alveolar spaces.
- Single tumor cells—scattered, free-floating cancer cells within the alveoli.

This classification was further refined [39,40] by distinguishing localized STAS (cell nests within three alveolar spaces from the tumor) from extensive STAS (nests beyond three alveolar spaces), and low STAS (1–4 clusters) from high STAS (5 or more clusters).

Diagnosing STAS accurately remains challenging, particularly in intraoperative settings. The accuracy of frozen section in detecting STAS remains a concern [41]. Frozen section was found to have high specificity (80–91%) for identifying STAS in stage I lung adenocarcinoma, but low sensitivity (44–55%), with an overall accuracy of 71% [42,43]. One major challenge in diagnosing STAS is distinguishing it from tissue processing artifacts—STAKS, which can mimic STAS on histopathologic examination. Previous studies have shown that neither percutaneous needle biopsy nor bronchoscopic biopsy performed before surgery in stage I NSCLC is associated with the presence of STAS [44]. Immunohistochemical staining and three-dimensional histological analysis have been proposed to improve the accuracy of STAS detection. Digital pathology and deep-learning algorithms are also emerging as promising tools for enhancing diagnostic consistency. However, diagnosing STAS accurately remains challenging, particularly in intraoperative settings. Substantial interobserver variability may arise when evaluating borderline or equivocal patterns, such as detached nests near the tumor edge or single clusters close to the slide margin. This variability reflects the current lack of universally accepted criteria for distinguishing true STAS from artifacts, leading to inconsistencies in pathological reporting across institutions. Intraoperative frozen section analysis, despite its high specificity, has persistently shown low sensitivity, making it unreliable as a standalone tool for surgical decision-making. These diagnostic limitations represent a major bottleneck in the clinical management of STAS and underscore the need for complementary, non-invasive predictive strategies. In this context, radiological and radiomic models may offer a valuable opportunity to anticipate STAS preoperatively, thereby supporting more informed surgical planning.

## 5. Surgical Perspective

Research has consistently demonstrated a strong association between STAS and poor prognosis in lung adenocarcinoma, especially in cases characterized by a predominant micropapillary growth pattern, which seems to show a higher proportion of STAS compared to other histologic patterns [45]. Much of the current literature has focused on early-stage tumors, where the presence of STAS has been linked to increased recurrence risk and worsened clinical outcomes. The reported rate of STAS in stage I lung adenocarcinoma ranges from 25.3% to 48% [2,46,47,48]. When including also stage II–III lung adenocarcinoma, STAS reaches a rate of 56% [15]: this finding reinforced STAS as a prognostic marker across all stages and suggested it may contribute to pathological upstaging. When considering only invasive mucinous adenocarcinoma, the rate of STAS increases to 65–72% [49,50], being a strong predictor of poor OS [51]. Among patients who underwent limited resections, STAS was significantly associated with higher rates of local and distant recurrence; however, this association was not observed in patients treated with lobectomy [2]. Even with a surgical margin of ≥2 cm, STAS-positive tumors demonstrate higher recurrence risks compared to STAS-negative cases [52]. This suggests that the standard margin recommendations for early-stage lung cancer may not be sufficient in the context of STAS. The margin-to-tumor ratio concept proposes that sublobar resections should be approached cautiously in patients with STAS to minimize recurrence. Analyzing patients with stage I adenocarcinomas, lobectomy provided better outcomes than sublobar resection in STAS-positive cases [46,53]. Similarly, STAS was found to be an independent risk factor for recurrence in stage IA part-solid adenocarcinoma patients undergoing sublobar resection [45]. On three-dimensional reconstruction, STAS tumor cells can adhere to the alveolar walls rather than appearing as free-floating clusters on two-dimensional sections [54]. This observation suggests that tumor cells detach from the primary lesion, migrate through the airspaces, and reattach to the alveolar walls, potentially via vessel co-option, which enables their survival and growth. Additional findings supported STAS as a predictor of recurrence-free survival (RFS) in stage pT1b/cN0M0, with recurrence risks comparable to stage pT2aN0M0 cases [55]. Notably, patients with stage IA and STAS showed similar outcomes to those with stage IB disease [47]: in tumors between 2 and 3 cm, STAS-positive patients had prognoses similar to stage IB, whereas tumors ≤ 2 cm showed no significant prognostic impact from STAS [56]. Systematic lymph node dissection also improved outcomes compared to limited dissection; in contrast, no significant differences in outcomes were found between surgical approaches or lymph node techniques in STAS-negative patients [46]. Histologically, STAS is not limited to lung adenocarcinoma; it has also been identified in lung squamous cell carcinoma, ranging from 19% to 40% [57,58,59], with higher stages showing increased STAS frequency. While STAS was linked to recurrence and poor outcomes in stage I, it did not show a significant prognostic impact in stages II and III, indicating that its relevance may vary with disease stage [58]. These findings established STAS as a significant prognostic factor for both distant metastasis and local recurrence in early-stage squamous carcinoma. STAS-positive patients had significantly shorter 5-year PFS compared to those without STAS [57]. Importantly, the researchers incorporated STAS into a prognostic nomogram, which showed potential in improving postoperative risk stratification in squamous cell lung cancer. Comprehensive analysis of STAS in lung neuroendocrine tumors (NETs) identified its presence in 26% of cases [60,61]. The incidence varied by subtype: 16–20% in typical carcinoids (TCs), 37–48% in atypical carcinoids (ACs), 43–71% in large cell neuroendocrine carcinomas (LCNECs), and 46–88% in small cell lung carcinomas (SCLCs). Among carcinoid tumors, the presence of STAS was significantly associated with higher Ki-67 proliferation index, lymph node involvement, and vascular invasion, reinforcing its role as a poor prognostic marker [61]. In NET patients, STAS was linked to early distant metastasis and a higher lung cancer-specific cumulative incidence of death, as well as it also emerging as an independent adverse prognostic factor for DFS and OS [62]. Furthermore, 28% of patients with multiple primary lung cancers (stages IA–IB) undergoing resection were found to have STAS [63], with strong correlation between STAS and increased recurrence risk, along with reduced OS, further supporting the negative prognostic implications of STAS even in early-stage multiple primary lung cancers. STAS was also studied in pulmonary metastasis. It was identified in 33–41% of patients with pulmonary metastases undergoing surgical resection [64,65]: the distance of STAS from the tumor was an independent risk factor for recurrence at the surgical margin. Additionally, patients with STAS had significantly lower OS and a higher risk of mortality compared to those without STAS, indicating a poorer prognosis. STAS was observed in metastasis originating from a range of primary tumors, including colo-rectal cancer, breast cancer, cholangiocarcinoma, and soft tissue sarcoma. Its incidence was higher in metastases from epithelial tumors than in those from mesenchymal origins. Current STAS-related guidelines are limited and primarily focused on invasive non-mucinous adenocarcinoma. Nevertheless, the presence of STAS in other tumor histologies has been increasingly documented. This observation highlights the need to refine existing recommendations by extending their scope beyond adenocarcinoma and by integrating evidence from diverse tumor subtypes. Developing such supplementary guidance could enhance the clinical relevance and applicability of STAS assessment across different histological contexts.

## 6. Chemotherapeutic Perspective

STAS is a well-established negative prognostic factor in NSCLC, highlighting the need for careful risk stratification and tailored treatment approaches in STAS-positive patients: focusing specifically on patients undergoing lobectomy, adjuvant treatment seems to improve prognosis in stage IB patients with STAS [66]. A large-scale analysis of more than 3000 patients with stage I lung adenocarcinoma was conducted to evaluate the role of adjuvant chemotherapy in the context of STAS [67]: the researchers recommended adjuvant treatments for STAS-positive patients with stage IB, as well as for those who were STAS positive with stage IA undergoing sublobar resection. However, chemotherapy was deemed unnecessary for those who underwent lobectomy. Another large-scale study on patients affected by stage I lung adenocarcinoma supported the use of chemotherapy in improving RFS in stage IB patients with STAS [68]. In contrast, no survival benefit from adjuvant treatment in stage IA patients was found, regardless of surgical method. On LCNEC, a subgroup survival analysis found that chemotherapy significantly improved both DFS and OS in stage I STAS-positive patients [62]. No survival benefit was observed in STAS-negative individuals, highlighting the potential value of STAS as a marker for adjuvant chemotherapy benefit in early-stage lung cancers.

## 7. Radiotherapeutic Perspective

Applying the concept of STAS to radiotherapy presents challenges, as its definitive diagnosis currently relies on histopathological evaluation of postoperative tissue specimens. Nonetheless, STAS’ presence has potential implications for radiotherapy planning. A positive preoperative bronchial cytology result—when combined with bronchoalveolar lavage or bronchial washing—indicating the presence of tumor cells in the air space, has been strongly associated with a high STAS burden [69]. While alone it cannot definitively confirm the presence of STAS, a positive result may reflect a high probability of its existence. Early-stage lung cancer patients with presence of tumor cells in the air space have shown worse regional failure-free survival and DFS compared to their counterparts without tumor cells in the air space, when treated with stereotactic body radiotherapy (SBRT) [70]. These results suggest that this status may serve as a useful predictor of treatment outcomes following SBRT in early-stage lung cancer. Indeed, STAS-positive tumors may require a wider clinical target volume (CTV) margin to account for possible microscopic extension into surrounding alveolar spaces. Consequently, adaptive radiotherapy protocols integrating radiomic and metabolic imaging features could improve target delineation in these cases. Additionally, incorporating STAS-related risk into multidisciplinary tumor board discussions could inform the selection of SBRT versus conventionally fractionated radiotherapy. Dose escalation or hypofractionation strategies could also be tailored based on predicted STAS burden, although prospective validation is needed. Future research should focus on integrating radiomic prediction models and pathologic data into adaptive radiotherapy workflows to refine dose delivery and minimize recurrence in STAS-positive patients.

## 8. Future Directions

As our understanding of STAS continues to evolve, several critical areas demand further investigation to translate current knowledge into improved clinical practice:

- Clarifying the biological mechanism: the underlying biology of STAS remains largely speculative. Future studies should focus on unraveling the molecular and cellular pathways that enable tumor cells to detach, survive, and migrate through alveolar spaces. Particular attention should be given to EMT, tumor–stroma interactions, and immune evasion mechanisms, including the role of tumor-associated macrophages and PD-L1 expression.
- Standardizing diagnostic criteria: variability in the pathological identification of STAS—compounded by interobserver differences and the potential for surgical artifacts—calls for standardized diagnostic protocols. Consensus guidelines should be developed to distinguish true STAS from artifacts, potentially incorporating 3D histology, immunohistochemistry, and AI-assisted digital pathology.
- Enhancing preoperative detection: frozen section analysis currently lacks the sensitivity needed for reliable intraoperative decision-making. Future work should refine imaging-based predictive tools, including CT feature analysis, PET/CT metabolic profiling, and advanced radiomic models. Integrating machine learning and deep learning algorithms may further improve accuracy, enabling personalized surgical planning before tumor resection.
- Integrating STAS into clinical staging and risk stratification: STAS is a strong prognostic factor but is not yet formally integrated into lung cancer staging systems. Risk models that include STAS, tumor size, margin distance, and molecular profiles could guide more nuanced treatment algorithms.
- Personalizing surgical strategies: emerging evidence suggests that lobectomy offers better outcomes than sublobar resection for STAS-positive patients, even in early-stage disease. Future clinical trials should assess whether segmentectomy, wedge resection, or more extensive surgery is optimal for specific STAS subtypes or locations (e.g., bronchiolar vs. alveolar STAS). The margin-to-tumor ratio should also be further validated as a surgical planning tool.
- Evaluating the role of adjuvant therapy: adjuvant chemotherapy appears beneficial in STAS-positive stage I patients, particularly after limited resection. However, optimal treatment regimens, timing, and patient selection criteria remain undefined. Prospective, STAS-stratified trials are needed to clarify the role of chemotherapy and radiotherapy across histologic subtypes and resection strategies.

By addressing these areas, future research can help establish STAS as a practical biomarker for surgical planning, treatment selection, and prognostication—ultimately improving outcomes for patients with lung cancer.

## 9. Conclusions

STAS represents a critical histological feature of lung cancer with significant implications for diagnosis, prognosis, and treatment. A multidisciplinary approach that integrates pathology, radiology, surgery, radiotherapy and oncology is essential to optimize management strategies for STAS-positive lung cancer patients. As research advances, the development of novel therapeutic interventions and refined clinical guidelines will be crucial in improving patient outcomes. STAS has been observed across multiple histological subtypes of lung cancer and is widely recognized as an independent adverse prognostic factor for both recurrence and overall survival. While the biological mechanisms underlying STAS remain unclear, current evidence strongly supports its clinical significance. Frozen section analysis, though highly specific, lacks the sensitivity for reliable intraoperative detection. In contrast, emerging CT-based radiomic models offer promising non-invasive tools for preoperative prediction of STAS and may help guide surgical decision-making more effectively. Continued research into the molecular pathways driving STAS, along with the refinement of predictive imaging and machine learning models, will be essential for improving lung cancer prognosis and tailoring treatment strategies. Future studies should focus on identifying the signaling mechanisms involved, refining risk stratification for STAS-positive patients, and integrating these insights into clinical practice to enhance patient outcomes.

## Abbreviations

The following abbreviations are used in this manuscript:

AC	Atypical carcinoid
AUC	Area under the curve
CT	Computed tomography
DFS	Disease-free survival
EMT	Epithelial–mesenchymal transition
FDG	Fluorodeoxyglucose
GGO	Ground-glass opacity
LCNEC	Large cell neuroendocrine carcinomas
MMP-7	Matrix metalloproteinase-7
NET	Neuroendocrine tumors
NSCLC	Non-small cell lung cancer
OS	Overall survival
PD-L1	Programmed death-ligand 1
PET	Positron emission tomography
SBRT	Stereotactic body radiotherapy
SCLC	Small-cell lung cancer
SMA	Smooth muscle actin
STAKS	Spread through a knife surface
STAS	Spread through air spaces
SUV	Standardized uptake value
TAMs	Tumor-associated macrophages
TC	Typical carcinoid
